# Space and place for health and care

**DOI:** 10.1080/17482631.2020.1750263

**Published:** 2020-10-25

**Authors:** Åsa Roxberg, Kristina Tryselius, Martin Gren, Berit Lindahl, Carina Werkander Harstäde, Anastasia Silverglow, Kajsa Nolbeck, Franz James, Ing-Marie Carlsson, Sepideh Olausson, Susanna Nordin, Helle Wijk

**Affiliations:** a Section for Nursing, University West, Halmstad, Sweden; b Department of Health and Caring Sciences, Linnaeus University, Kalmar, Sweden; c Department of Cultural Sciences, Linnaeus University, Kalmar, Sweden; d Faculty of Caring Science, Work Life and Social Welfare, University of Borås, Borås, Sweden; e Department of Health and Caring Sciences, Linnaeus University, Växjö, Sweden; f Institute of Health and Care Sciences, Sahlgrenska Academy, University of Gothenburg, Gothenburg, Sweden; g Sahlgrenska Academy, University of Gothenburg, Gothenburg, Sweden; h Academy for Design and Crafts, University of Gothenburg, Gothenburg, Sweden; i School of Health and Welfare, Halmstad University, Halmstad, Sweden; j Institute of Health and Care Sciences, University of Gothenburg, Gothenburg, Sweden; k Centre for Ethics, Law and Mental Health, Gothenburg University Hospital, Gothenburg, Sweden; l School of Education, Health and Social Studies, Dalarna University, Falun, Sweden

**Keywords:** Space, place, health, care, transdisciplinarity

## Abstract

**Purpose**: This discussion paper aims to contribute to a greater understanding of the state of the art of research engaged with conceptual matters of space and place for health and care.

**Method**: The authors, who represent a variety of academic disciplines, discuss and demonstrate the conceptual recognition of space and place in research in health and caring sciences building upon own work and experience.

**Results**: To explore the concepts of space and place for health and care is a research pursuit of utmost importance, and should be made through transdisciplinary research collaborations, whereby spatial theories from various disciplines could be communicated to cultivate truly novel and well-informed research. Furthermore, engaging with relational and topological perceptions of space and place poses methodological challenges to overcome in future research on health and care.

**Conclusions**: We argue that there is a need for accelerating spatially informed research on health and care that is informed by current theories and perspectives on space and place, and transdisciplinary research collaborations are a means to achieving this.

## Introduction

This discussion paper is the outcome of both the shared experiences and diverse understandings of the authors, who together represent a variety of academic disciplines, but who nonetheless agree that problematizing and operationalizing the concepts of space and place in health- and care-related research is essential. As a research network comprising researchers from fields like public health, human geography, architecture, design, sociology, reproductive health, and caring science, we seek to discuss and promote the value of a more multidimensional understanding of space and place for health and care.

Space and place are general concepts that are widely used also in the health and caring sciences. However, we believe that there is a tendency to take them for granted and apply them to matters of health and care in ways that do not explicitly consider the broader range of conceptual and theoretical alternatives that exist within the human and social sciences. For example, in contemporary theorizing place and space are most often understood in relational and topological terms, as denoting something actively involved in human, as well as non-human, becomings. This is quite different in comparison to previous times when both place and space more denoted a passive backdrop for human social life and functioned as passive containers, or frames of reference, from which matters of health and care transpired.

How we understand and use the concepts of place and space thus have important implications for how we understand care and health. If we, for example, conceptualize space (or place) as merely a “somewhere”, a location on the Earth´s surface, then it also becomes little more than a passive background for health and care. However, if we instead conceptualize place (or space) as active, then it becomes participative in the very making of care and health. This illustrates that the concepts of space and place are crucial for the development of knowledge of, and for, health and caring. Consequently, it is of great importance for the advancement of the health and caring sciences. This aligns with a disciplinary goal that has been pursued at least since Andrews (2003) stated the clear potential for interdisciplinary research on space and place for health. Later, Andrews further recognized the evolution of a nursing research explicitly concerned with spatial aspects and geographical approaches over the preceding 20 years (Andrews, 2016b).

We argue that future research on space and place for health and care should be pursued further, and put into practice through transdisciplinary research collaborations whereby theories of place and space from various disciplines could be communicated across academic borders to cultivate truly novel and well-informed research. We also stress the urgent need to accelerate the research process with regard to space and place for care and health. It is our belief that by making explicit various perspectives, and discussing the ways in which different ideas related to space and place could contribute to a better understanding of health and caring, the knowledge field of space and place for health and care can be expanded. More practically speaking, this exploration could also contribute to improving health and caring actions, as well as institutions and organizations. To explore the concepts of space and place for health and care is, we believe, a research pursuit of utmost importance.

Consequently, the aim of this co-authored discussion paper is to contribute to a greater understanding of the state of the art of research engaged with matters of space and place for health and care. We will draw upon our own experiences of theoretical and methodological challenges, e.g., discrepancies, confusion, and feelings that something is missing or inadequate in the way we think or work. We do so by first reflecting on theories of space and place, and a few other spatial concepts, in relation to health and care. Then, we move on to some of our experiences from conducting this type of research, specifically where confusions, conflicts, and thought-provoking situations have occurred. In the latter part of the paper, we contextualize and illustrate theoretical underpinnings and our experiences of new ways of thinking. In the last part, we offer a set of tentative points and suggestions for further discussion and summarize our main arguments.

## Space and place—theoretical and conceptual underpinnings

The members of the research team behind this paper not only represent various academic disciplines but also embody different perspectives on health and care, as well as on space and place. These perspectives build, at least in part, on different theoretical underpinnings and disciplinary trajectories. We consider this a strength, but also recognize it to be a challenge that underscores the importance of articulating and discussing these underpinnings. Below we illuminate a few of the spatial perspectives and concepts we have encountered in our research team in the context of the caring and health sciences.

### Geographical perspectives in caring science

In caring science, “environment” is considered a fundamental concept, along with “human”, “health” and “caring” (Cutcliff & McKenna, 2005). Environment has a conceptual history of its own, but for us it has opened the door to spatial aspects of care and health. In turn, this has led us to the concepts of space and place, because these have commonly been used when researching such aspects. In retrospect, we ask ourselves if this translation, or delimitation, of “environment” into place and space also may make other parts of health and care less visible, for example, the biological parts of the environment.

In caring research space and place have often been tacitly recognized also under “geographical perspectives”, dating back to Nightingale´s *Notes on Nursing: What it is and What it is Not* from 1859, in which the importance of health-care settings was emphasized (Andrews, 2003). However, it seems to us that over time geographical perspectives, here ´settings´, have increasingly been narrowed in caring research, in effect leaving out, say, geographical conditions of the environment like weather and vegetation. This can be exemplified by Liaschenko’s (1994, 1997) research from the mid-1990s on home care and nurse–patient relationships, which entailed a wider recognition of the importance of considering spatial dimensions. Specifically, Liaschenko (1996) extended the argument that the understanding of place is important in all nursing research, as different places and institutions do different kinds of work, involving different values and being structured by different philosophies. Liaschenko (2001) also highlighted the need to reflect on nursing geography in nursing research, arguing that nurses often take their empirical geographies for granted and seldom reflect on how their work is affected by location and geographical distance. Also, Malone (2003) raised awareness of the fact that all humans are spatial beings, and that spatial aspects of their lives include familiar routines that also contribute to their identities. For example, when a person is ill, he/she could be displaced from these everyday time–space routines, as when leaving home and entering the unfamiliar environment of a hospital. The private space of the home is thereby also lost, together with the privacy of one´s own body as it becomes accessible to others in an institutional space. In such spatial circumstances, patients turn to nurses for support.

Building on our own research, as well as clinical caring experiences, we identify that one challenge in caring is for staff to have knowledge about spatial aspects and place factors that contribute to a healing and enriching environment for patients and relatives. The caring science perspective permeates caring research, education, and clinical practice. It also entails the responsibility to improve caring practices and behaviours. To this, we would like to add that all caring situations are somehow tainted by space and place. Consequently, we believe that caring/nursing geography has the potential to further inform clinical practice about how the spatiality of caring environments (physical, relational, human, non-human) can be improved.

### Health, caring, and geographical scale

When exploring space and place in relation to health and care, we have encountered the question of scale, including the argument that both macro and micro perspectives should be considered. Macro perspectives focus on such issues as the organization of health-care systems and social work, as well as on architecture and design for human health. Such perspectives would argue that these systems and structures must be sustained and developed whenever and wherever needed.

It should be mentioned that some topics from a macro perspective now include issues at planetary scale, such as climate change, loss of biodiversity, and the societal and political responses to the climate crisis. The planetary scale is central in the transdisciplinary field of planetary health and is partly an alternative to the global scale that underpins the field of global health. Yet, research on place, space, care, and health of course also entails the lived experiences of individuals and/or groups, which necessitates experiences with micro-level interactions as well as knowledge of philosophies relevant to a local scale. Overall, this suggests that it is important to situate health and caring in relation to different geographical scales, and we would recommend that we draw upon competences and knowledge from a wide range of fields and disciplines.

As an academic discipline, caring science is based in human science and in existential philosophical traditions, but the field is not dedicated or related to a special health profession per se. However, caring is the foundation of professional nursing, supporting the notion that the provision of good care will most certainly support better health. However, the concept of health, according to the definition given by the WHO (1948), can also be understood in a wider context: “Health is a state of complete physical, mental and social well-being and not merely the absence of disease or infirmity“ (WHO, n.d., para. 1). This multidimensional definition highlights three aspects of health: the physical, the mental, and the social. Furthermore, it emphasizes that health should be understood as something more than just the absence of illness, in addition to promoting the view that health is something individually experienced. The definition is silent on space and place, and therefore invites a more transdisciplinary approach on such matters, as well as a recognition of the variety of geographical scales that are involved in the different and intertwined dimensions of health matters and how they can be improved (Lyckhage et al., 2018).

### Life-world—recognizing space and place

In the health and caring sciences, it is common to understand health and care through the phenomenological concept of “life-world”. However, when Van Manen (2014) used “life-world”, he included not only existential or human experience of relations, corporeality, but also spatiality, temporality, and materiality (i.e., things/technology). This means, especially in relation to “spatiality” and “materiality”, that it is possible to conceptualize the life-world so that it also incorporates space or place. The life-world of care and health always takes place somewhere. Accordingly, the concept of “lived place” can include the physical context for health, for example, the materiality of a place and its material infrastructures. “Lived place“ also takes social norms into account, how people attach meanings to health and place, and how societal organizations manage and influence health-care institutions.

We have also come across a similar concept in our research, and that is the concept of “lived space”. Although it is sometimes used to refer to how people in a more restricted sense experience the spatiality of a room (Andrews, 2003; Lindahl et al., 2011), it is most often understood in a way quite similar to “lived place” (Lefebvre, 1992). This illustrates that space and place are concepts that are sometimes used interchangeably and sometimes they are given different meanings. This has been a source of confusion for us, but also reassured us that we should continue searching for ideas behind the concepts.

When it comes to place and space, we have learned that these concepts have in fact often been contrasted and understood as opposed. For example, in the development of health geography (a sub-discipline of human geography) in the 1990s, place was promoted as an alternative to space because it provided a better account of the human dimensions of health. Place was viewed as something that people filled with meanings and feelings, and in that sense, one root of health geography goes to phenomenological philosophy. Space, on the other hand, was often conceived as merely marking a physical location or a concept that mapped only the visible features in terms of spatial distances and distributions. This idea of space is basically a three-dimensional Cartesian-Newtonian construct, one by which any physical entity can be mapped by the coordinates of width, length, and height. On that theoretical/philosophical basis, one can then identify (mark) the space in which care occurs, but not the human meanings, sufferings, or joys related to health (Andrews, 2003).

In our research group, we have identified that this ´container view of space´ is still often used in research on health and care, even though there are now other views that embrace relational and topological aspects, and offer other analytical tools (see below). This insight has also caused some tensions within our group, especially in some of our initial work. Being fostered within the frameworks of different disciplines, we entered the collaboration with different knowledge about spatial theories and all the concepts that come with them. It is relatively easy to agree that place and space matters for health and care on a general level, but more difficult when it comes to the theoretical and philosophical underpinnings. We have also experienced it to be somehow difficult to articulate, and get support for, our ´space and place agenda´ when communicating theoretical and methodological challenges to forums outside the research group. It has gradually become clear to us that any disciplinary tradition that uses a certain set of theories and methodologies can also stand in the way for a further development and usage of alternative concepts and ideas. For us, that experience also underscores the need to develop ways of communicating about theories on space and place, as well as their underpinning ideas, between researchers working across the fields of health and care.

### Towards relational, topological, and becoming spatialities

The life-world concept touch upon spatial ideas, such as relations between humans and non-humans, and between a corporeal individual and its material/physical surroundings. This has opened up an avenue for us into other sets of ideas that are also related to the concepts of space and place, and which we have tried to operationalize in our own research. We argue that this opens up for spatial perspectives that not only should be taken into account but also challenge us to reconsider and perhaps revise theoretical concepts, theories, and models used in research on health and care. Here we begin with the environment concept in a spatial perspective.

The Norwegian nursing philosopher Kari Martinsen (2006) metaphorically described the atmosphere in health environments as a song that could be sung in various tones and sounds. In caring environments, the song is reminiscent of a hymn or a praise to the surrounding world. The opposite, when the environment is marked by sorrow, pain, or insults, converts the song into one of melancholy, tears, or even screams (Martinsen, 2006).

From a phenomenological perspective, to be orientated means to be turned towards or away from what is there (Ahmed, 2006), e.g., care and change. As we understand it, also atmospheric health environments can be regarded as vital non-human agents that affect not only the obvious and intentional but also one’s sense of health when being in the world. The concept of place has, especially in its phenomenological tradition, been understood in a similar way. Place is more than just a frame for individuals’ reality and experiences (Tuan, 1979), and a *sense of being* means to dwell in a place, being somehow anchored and rooted (Cresswell, 2014). In other words, place is here ascribed an agency that ´provides cues for our behaviour, varies within individual and cultural groups’ (Tuan, 1979, p. 389). Furthermore, place becomes a constituent in the formation of identity, as it provides ontological security and emotional attachments (“topophilia”) to our being in the world. This conceptualization means that place, like atmospheric health environments and their spatialities, is an intimate part of how we become human, and what characterize us as human spatial beings.

We have also encountered a parallel theorization of space. This has included a shift from absolute to relative conceptions of space, and, more recently, an engagement with relational, topological, and non-representational understandings (Andrews, 2016a). One of the approaches found under the umbrella of relational theories of space is non-representational theory (NRT). The theory was first developed in the late 1990s in human geography and has since spread and evolved in many other fields and disciplines (Andrews, 2016b, 2018). NRT can be understood as an ontological approach that aims to animate the active lived world, the taking-place of events, the becomings, the movement-space(s), rather than to map what has already happened (Anderson & Harrison, 2010; Andrews, 2018; Vannini, 2015). Consequently, it is also an approach that seeks to reveal how the present might, or could, take other directions and thereby NRT aims to open up the world to other possibilities.

NRT is directed towards the pre-cognitive realm and the affective domain, i.e., the residuals of what representational thought often leaves aside. Interestingly enough, this includes fluid atmospheres, and we have here identified a conceptual overlap with atmospheric health environments. We also believe that NRT corresponds to an embodied understanding of space and place that, arguably, is close to the core concerns in caring, nursing, and health science. NRT ties in with a more general “spatial turn” in the social sciences and humanities, and contributes to opening up health research towards various social science directions, and disciplines such as human geography (Andrews, 2016a). The development in theorizing space and place in the last 20–30 years, and the results thereof, is, we argue, something that must be considered, as well as put into the heart of health and care research practice. Although some researchers have already made great contributions in this regard, our experience is that it has not yet expanded enough into the health and caring sciences. These are disciplines where matters of health and care constitute the core, and if being spatial is a part of what it means to be human, then it has everything to do with health and care.

## Some examples and experiences from our own research

The following examples aim to provide further contribution to the discussion on the relevance of space and place for health and care. They show how we in research have encountered and struggled with spatial aspects and some of the central thoughts that have emerged among us. They also mark some moments that eventually coalesced into this discussion paper.

### Researching institutional care environments

Much research and studies on health and care relate to some kind of institutional care environment, such as hospital wards or residential care homes. This is also an active research field among several members of the research team and has been the subject for reoccurring reflections on spatial issues. One of these that we have struggled with is that space and place are not only concepts that we use in research, they also appear like objects ´out there´.

The first example is from research conducted in residential care, a form of care and living that affects many people. Currently, around 82,000 older persons are living in residential care facilities in Sweden (National Board of Health and Welfare, 2018). In these facilities, the common communal spaces are the primary locations for activities and social interaction. Research has shown that older persons living in residential care facilities in Sweden spend most of their time in their private rooms or apartments, isolated from each other (Bowie & Mountain, 1993; McKee et al., 1993; Nordin et al., 2016; Ouden et al., 2015). This building design reﬂects a period around 30 years ago when older persons living in care facilities were in better health and were more mobile compared to the resident population of today, and were thus able to spend more time in communal spaces interacting with others. This is one aspect that explains why older persons with frail health do not leave their apartments. Another aspect is that although communal spaces are part of the older persons´ living environment, they do not belong to the individual. According to Andersson et al. (2014), there is a clear demarcation between the private apartments and other, more public spaces in the facility, and older people describe the apartments in terms of their homes (Andersson et al., 2014). This may be comparable to Martinsen’s description of the patient room as a place that can provide boundaries and give security and protection, and by that allowing space for something that in many ways could be beyond comprehension (Martinsen, 2006).

In our research on residential care facilities, several spatial aspects and questions arose. How should the role of the build environment, its design and materiality, be conceptualized as active spatial parts in the care and health of the residents? Another question was about boundaries between private and public space in relation to safety and security. We also identified a tension in the usage of place and space concepts. On the one hand, they often appear as names for things that are almost like objects, i.e., a patient room or communal building we can go to. On the other hand, both place and space are also often used in order to signify social demarcations and boundaries that are beyond the physical or material. At times this dual character of space and place has been a source of confusion, and we also believe that it can act as a hindrance in communication. We have also learned that space and place in the social sciences and the humanities are most often used in a social constructivist sense, which means that there is some social-spatial dialectics at play. This can also be the case in caring science, although not always explicit as our next example illustrates.

One model in caring science that includes spatial aspects is the Ecological Model of Ageing. This model defines a person as having a set of competencies (e.g., physical and cognitive health) and views the environment in terms of demands or pressure (e.g., barriers in the environment). When there is a balance between the person’s competencies and environmental pressures, positive outcomes can occur, whereas a mismatch can result in negative outcomes. With increasing levels of frail health (e.g., cognitive disabilities and dementia), there is a risk that pressure from the environment will become too severe in relation to the person’s competencies (Lawton & Nahemow, 1973).

In the perspective of this ecological model of ageing, engaging in activities and social interactions is essential for older people with frail health (Lawton, 1994), and can contribute to their perceptions of well-being and the meaningfulness of daily life (Rowles & Bernard, 2013). The national fundamental values of elderly care in Sweden state that the goal is to ensure that older people can live a dignified, comfortable, and meaningful life with the opportunity to socialize with others (National Board of Health and Welfare, 2012). Although not explicitly stated, we read this as a socio-spatial problematic of health and care. These are also to be found in relation to environmental factors, which play an important role in person-centred care (McCormack & McCance, 2006). They have the potential to ensure well-being (Nordin et al., 2017) and independence for people with frail health (Brawley, 2001), enhancing their possibilities to participate in activities and social interactions (Barnes, 2002; Joseph et al., 2015; Nordin et al., 2016). As the individual’s health declines, the physical environment should be adjusted to accommodate this change (Lawton & Nahemow, 1973). This also illustrates the importance of not forgetting or taking the body for granted when place and space are conceptualized. What may be a perfect space for one body could instead be a set of obstacles for another.

According to the given examples of research related to institutional care environments and the ideas provided by the model above, one’s surroundings have an impact on health. What we would like to highlight is that the socio-spatial ingredients in these environments and surroundings, and hence their consequences for health and care, are here more indirectly articulated and addressed. This observation has, again, pointed us in the direction of delving further into the spatial ideas underlying theories, concepts, and models that are applied in research on the environment for health and care.

### Researching home

One central concept used in research on health and care is ´home´, which evokes many ideas about space and place. In our research, we have encountered some of them. In one theorization the concept of home is defined by three dimensions (Oswald & Wahl, 2005): (1) the social home—where several actors interact across the home space—is especially explicit when home care is an issue; (2) the physical home—layout, furniture, pictures, cleanliness, location; and (3) the emotional home—how people connect to their internal home space and their external community environment and neighbourhood. Milligan (2005) highlighted that emotions and personal identities are deeply embedded in ideas about home, while Blunt (2005) showed that these dimensions interact, thereby also demonstrating that the meaning of home is dynamic, changeable, and concerned with lived experiences, social relations, and emotional geographies.

Home is often depicted in an idealized way (Lindahl et al., 2011), such that the home constitutes the private space and a place for personal growth and everyday life. Home provides the freedom to live according to one´s own routines and preferences (even if a home can also be one of the most dangerous and unsafe of places, e.g., abused women). The experience of home is even described as a process of integrating the self with the physical, social, temporal, and spiritual environment. One’s relationship with family and friends enables a social connection and thus promotes a sense of at-homeness (Molony, 2010; Roush & Cox, 2000). However, if the spatial prerequisites do not allow integration between a person and the environment, experiences of an existential homelessness, confinement, restriction, and powerlessness may occur (Molony, 2010).

When researching home we have come to realize the importance of thinking geographically. Where is the home that we are researching? For example, in developing countries, there among Sweden, care and health work have increasingly moved spatially from hospitals and other health-care institutions into peoples´ own homes. The moral consequences of this changed geography, when professional care moves back to the home from the hospital, have implications for patients, families, and nurses (Liaschenko, 1994, 1997). Furthermore, it raises issues about space and place that are often taken-for-granted. There may be an invisible and un-reflected shift in power-relations when care is performed in the home space concerning decision-making, intimate relations, and privacy. Caregivers providing care in private homes may encounter challenges since their workplace conditions typically diverge from those experienced in another person’s home. Caregivers lose, in a way, their control of the health-care environment while simultaneously being tasked with ensuring a safe home care environment (U.S. National Institute for Occupational Safety and Health, 2010).

In relation to health-care professionals, we have come to believe that it is important that they have knowledge of the spatial aspects of the work they are responsible for. Such knowledge would provide them with useful tools for how to consider, act, and perform health and care work under various spatial conditions, for example, in private homes.

### Health and care in the context of socio-spatial power relations

Questions that have to do with power have kept many geographers, political scientists, historians, sociologists, and others busy for a long time. In our research team, we have encountered them not only in relation to health and care but also in relation to place and space. Power does not only exist in the social world of ideology and politics but also in material and spatial forms.

This means that power manifests itself in the social-spatial structures of health and care. This could be in the form of ideology and authority structures within health and caring institutions, in the caring contexts of the built environment, or even down to how the bed and specific technological equipment are spatially positioned (Poland et al., 2005). The implication of a lack of power of, and over, space has been understood as alienation and disempowerment, as well as in terms of being out of place and place-lessness (Lock & Gibb, 2003; Nilsson, 2014; Seibold et al., 2010). In relation to care and health, Poland et al. (2005) argued that power is exemplified by who sets the agenda and whose interests are served. These factors must be made visible in discussions and debates on how care is performed, and how health is constituted somewhere. As we would argue, and in line with social constructivist theorizations of space and place, power relations and power constructions should be conceptualized as being socio-spatially present wherever care and health take place.

### Spatial aspects of medical technology

Medical technology is an embedded and active part of health and care environments, however simple or complicated it may be. An obvious example is a needle, which clearly can affect a caring situation, also in terms of expectations, fears, and trust. Studies conducted in advanced home care show that knowledge about the various technological devices in use in the home, e.g., ventilators, suction devices, and nutrition pumps, is essential for establishing a feeling of confidence. In contrast, studies exploring parents’ lived experience when having a child with ventilator treatment have revealed that the essence of home—that is, a space or place that typically is signified as private—has changed due to the presence of technology and the professionals who work in the home (B. Lindahl & Lindblad, 2011, 2013). Many parents have stressed the importance of letting the “right one” into your home, which also points to the importance of socio-spatial boundaries. In cases where relationships and knowledge about technology are sufficient, a caring relationship can be established between the child/young person, professionals, and the family, which in turn can contribute to extended growth, well-being, and a comforting atmosphere (Israelsson-Skogsberg et al., 2018; Israelsson-Skogsberg & Lindahl, 2017; Martinsen & Dreyer, 2012).

### Space and the intensive care unit

An intensive care unit (ICU) is a complex space. It includes the presence and use of a spatial arrangement of a large number of non-human medical technologies and is further characterized by medical treatments, high workload and staffing, patients’ illness and vulnerability.

There is an old spatial pre/conception that patients cared for in the ICU are not at all aware of their bed spaces or the spatiality of their surroundings. However, today, pharmaceutical treatments and drugs have gone through tremendous development, and patients now often receive a light sedation regimen (Egerod et al., 2015; Holm & Dreyer, 2018). This means that these patients are conscious and thus more spatially aware of their surroundings. However, this fact has not yet been fully considered in the design and building of patient rooms. In addition, spatial issues that concern patients´ loved ones have often been totally ignored. An ICU is also a closed space hidden from view from others in order to protect patients’ integrity. In some ways this intensive care space resembles what Levinas (in Kemp, 1992) called a “secret place”.

In research that has considered the spatial aspects of ICUs, some emphasis has been placed on the physical environment. There are several active non-human components that constitute the environment in an ICU, such as sound, cyclic light, interior decoration, placement of technology, and also human views of nature (Johansson, Bergbom, Waye et al., 2012). At an ICU, there are few differences in sound levels between night-time and daytime (Johansson, 2014; Johansson, Bergbom, Lindahl et al., 2012), as treatment, care, and staff conversations are performed around the clock. However, even though sound levels have been found to be high in ICUs, they could easily be improved through the use of sound absorbents behind walls and ceiling. When a patient room in an ICU was refurbished according to the principles of evidence-based research (Ulrich, 2006), and in order to communicate a song of caring and praise, restorative energies were conveyed to patients and their loved ones as well as to staff (Lindahl & Bergbom, 2015). The results showed that both patients and visitors evaluated the cyclic light system as comfortable (Engwall et al., 2014, 2015).

When engaging with contemporary approaches to space and place, for example, NRT, we have realized that ´non-humans´ (or ´more-than-humans´) are now often included. Medical technology, as part of the caring environment, is thus one of those non-humans that provides an agency that is very significant for care and health. However, in the health and caring sciences it remains a challenge to theorize medical technology as a non-human spatial agency. One particularly important spatial aspect is that technology enables care and health actions to operate over distance.

### Researching carceral design and care

The design of and for care and health also raises spatial aspects. Indeed, carceral design can be understood as a powerful agent for spatially coordinating humans by, for example, controlling and orientating the body in a specific direction. Carceral care settings are institutional spaces built for surveillance and control, but also aimed at facilitating care-giving, which is somewhat paradoxical given the negative consequences that incarceration often conveys (Hammerlin, 2018; Sommer, 1976). Some of the places and spaces in carceral care settings, such as forensic psychiatric hospitals and special residential youth homes, are aimed at fostering care that should lead to some kind of change.

For children and adolescents (youth), this transitional change refers to a life free of criminality, drug abuse, and anti-social or self-destructive behaviour. For patients in forensic psychiatric care, change is the (re)habilitation from their so-called ´mental illness´, and for the youth at the special youth homes, it is change from severe problems related to criminality and drug abuse. The fact that troubled youth and patients are essentially incarcerated, and given compulsory care, raises several questions as to how their lived experience, health, and well-being are affected by the spatiality of the design of their everyday place and space. Incarceration, which relates to both physical and mental restraints, affects and limits the *spatial, relational, corporal, and temporal lifeworld* (Van Manen, 1990).

Furthermore, incarceration inevitably brings about a loss of freedom, social relations, power, economic standard, progression in life, and cultural development (Hammerlin, 2010). Special residential youth homes and forensic psychiatric hospitals are institutions built upon layers of carceral architectural heritage rooted in asylums and prisons from the 19th century. They are commonly constituted by an interior carceral design, e.g., hard materiality, simplified objects, and fixed furniture that aids surveillance. Features such as high walls, locked windows, lockable steel doors, and security cameras are designed to produce docile bodies (Foucault, 1991) that are obedient, ready, and trained to be examined and rehabilitated, as well as to obey the rules of the institution in the hope of eventually being released. The incarcerated body is thus formed spatially by the carceral, and conversely, it forms the carceral by resistance, e.g., violence as empowerment against confinement (although some bodies become obedient, docile, etc., and do not resist). Given all this, one may ask if it is even possible that carceral care settings can support care towards the achievement of the desired change? More generally, we have come to realize that space and place, as concepts and as empirical phenomena, are double-edged characters. They can serve both the purposes of imprisonment and the emancipation of care and health. Yet, it may be that one of the characteristics have often has the upper hand.

James and Olausson (2018) explored meanings of place and space for incarcerated youth from an existential point of view (and employed van Manen’s existential *lived space* as a departure point). Their findings showed that the meaning of place through the youth’s perspective could be understood as *being disempowered and fighting for dignity*. Similarly, patients’ narratives display how the physical limitations of space, in forensic care settings, become a place of discomfort and challenge the maintenance of dignity, identity, and everydayness (Olausson et al., 2018). Moreover, being incarcerated can be experienced as time standing still while the outside world moves along. Places for incarceration, i.e., spaces organized through a spatial network of objects with carceral design, can have adverse effects on the body by inflicting corporal pain and discomfort (James & Olausson, 2018; Olausson et al., 2018). It is not unrealistic to assume that carceral care settings, per se, make youth and patients less susceptible to care and hinder the desired transition. Together, these findings point to not only specific meanings produced by place and space but also to how places of incarceration risk undermining the essence of care. In turn, this illustrates that space and place can do things with (and to) people while also exemplifying perspectives on the relationally between space, place, health, and well-being.

## Discussion

The examples and reflections in previous sections were selected from our own research and academic engagements with the spatial aspects of health and care. At times, these were already present from the very beginning, but they also emerged and increased in importance during our individual and collective research processes. In fact, we believe that we have encountered the real difficulties when writing this discussion paper. Not only has this forced us to confront our own broad range of spatial ideas, theories, and varied research interests, but also how to represent these in a coherent form that at the same time reflects internal variation and our inconsistencies.

We have struggled with the concept of space and place, but also with how they reverberate with other concepts that also express spatial ideas, such as landscape, atmosphere, and room. For example, it is possible to understand space and place through the lens of the room as *being* the room for health and care (Werkander Harstäde & Roxberg, 2015). We could also have paid more attention to the concept of landscape. It can capture more of the natural features and environmental factors of importance for health and care, and the `therapeutic landscape` is of course a concept that has already been widely used. Or, for that matter, we could have engaged more with geographical features and conditions that sometimes get buried underneath the space and place of health and care. Nevertheless, this has reinforced our main argument that matters concerning space and place should be considered in all areas of research on health and care. This, we believe, requires some kind of transdisciplinary approach with an aim of reconsidering and revising traditional or common models and theories in health and caring sciences in light of space and place matters.

As mentioned earlier, and as Andrews (2016b) pointed out, nursing research is beginning to embrace recent geographical and spatial approaches—however, only in small steps. This became apparent in a recent analysis of ideas related to the concept of home in palliative care research, an analysis which included an exploration of revealed spatial expressions related to home as a concept (Tryselius et al., 2018). The concept analysis showed that even though “home as actor” was the most common attribute (acknowledging active, relational, and topological perspectives of space/place), its meaning was not further specified or developed in relation to such thoughts (Tryselius et al., 2018). Moreover, in attributes communicating the concepts of space, place, and environment, the expressed spatial perspectives were found to relate to relational geographical thoughts in a very limited way (Tryselius et al., 2018). The authors found spatial expressions related to the concept of home which explicitly included relational conceptions of space and an ontology of becoming in only one of the 16 analysed articles (Tryselius et al., 2018). The results in the study thus demonstrate, on a sub-discipline level, the story of geographies of nursing that Andrews (2016a) has been writing.

To this we can add that we have thought about the importance of language in relation to how we formulate ideas about space and place in words. In order to express an ontology of becoming there are reasons for being cautious when using prepositions and definite forms. For example, ´*in* space´ and ´*the* place´ are often tied to an absolute or Cartesian-Newtonian conception of space and place. If we instead would like to convey relational conceptions of space and place, then we might have to use verbs like ´spacing´ and ´placing´. However awkward they may sound, they do more adequately express processes and becomings.

Drawing to a conclusion, we argue that space and place and their relations to health and care must be more systematically considered as important theoretical and conceptual underpinnings in the health and caring sciences. Consequently, we also believe that the development and implementation of spatially informed and concerned research on health and care ought to be accelerated. In the following, we propose our ideas and suggestions as food for thought and for future discussions on the topic. In order to further develop and accelerate a more spatially engaged and informed research on health and care, we need to:

- develop ways of communicating spatially related sets of ideas, as well as theories on space and place between researchers working within the field of health and care;
- decide on who should be responsible for setting the agenda and whose interests are to be served for further development (many people are involved, and on many different scales, in matters regarding health and care—patients, families/relatives, staff of all kinds, designers, builders, leaders, politicians, among others);
- understand and integrate our results into practice;
- reconsider and revise theoretical concepts, theories, and models, and to create new or updated versions;
- overcome methodological challenges when engaging with relational and topological perceptions of space, as well as spatial ideas relating to an ontology of becoming;
- equip health-care staff sufficiently with evidence-based knowledge about space and place, for the benefit of patients and workplace health;
- improve nurses’ knowledge about what constitutes a healing and enriching environment in health care;
- inspire the implementation of evidence-based knowledge to influence the future design of health care.

## Conclusion

The tentative list above reflects our belief that spatial considerations and knowledges, which in one way or another are significant for health and care, must be central for the health and caring sciences. When it comes to the implementation of such a place-space agenda, our experience is that it would be best achieved in transdisciplinary research collaborations. Apart from being knowledgeable in contemporary theories of relevance in a space-place perspective, it is also important to understand their underpinning ideas and where they come from. We would also add that an interest in research on space and place, related to health and care, should also be cultivated among potential fundraisers.

This discussion paper stems from our own experiences from researches and collaborations. We have tried to share some of them, as well as our reflections on the concepts of space and place in relation to health and care. The paper has been written with the aim of contributing to an understanding of the state of the art of research engaged with matters of space and place of relevance for health and care. As authors we are not altogether sure that we fully agree on everything said, nor are we certain that we share a common understanding of the proposed place-space agenda for the health and caring sciences. Nevertheless, in summary, we are in agreement that there is an urgent need for developing research on health and care that is more systematically informed by various contemporary theories and perspectives on space and place, including other neighbouring relevant spatial concepts.

We think that the best way to develop such research is to work in transdisciplinary research collaborations, in and through which ideas and theories on space and place from various disciplines can become more easily communicable and also put into creative novel research practices. Thus, in order to accelerate the pace at which spatially oriented research on health and care is conducted, and to overcome the challenges we have listed (above), we strongly believe that there is a great need for a future increase of transdisciplinary research on health and care. This increases the possibilities to gain a richer understanding of space and place concepts, ideas, and theories that are often interrelated in complex ways and across disciplinary boundaries. A transdisciplinary approach would also be beneficial for the communication about these matters, both internally and externally. We also believe that it provides a platform for designing and conducting well-informed, and perhaps even ground-breaking research. We are actually quite convinced ourselves that a transdisciplinary place and space agenda could contribute significantly to the development of health and caring sciences.
